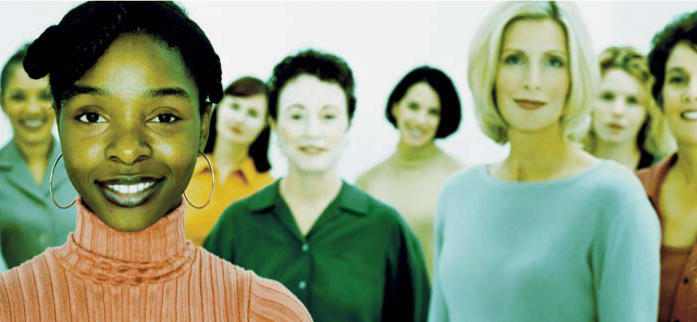# Making Progress on Breast Cancer

**DOI:** 10.1289/ehp.114-a98

**Published:** 2006-02

**Authors:** Luz Claudio

For the last two years, scientists across the country have been working together in a highly collaborative effort to uncover the links between exposure to environmental pollutants, puberty, and development of breast cancer. On 10–11 November 2005 the Breast Cancer and the Environment Research Centers (BCERCs), a joint effort of the NIEHS and the National Cancer Institute, came together in Michigan to share what they have accomplished to date. Each center reported on advances made over the past year in their two research components: 1) research on the basic biology of mammary gland development, and 2) epidemiologic studies of how environmental factors affect puberty in girls.

The impetus for creation of the centers came from appeals from advocates, who urged the NIH to support a more comprehensive approach to research on the environmental causes of breast cancer. “We wanted a research approach that focused on environmental causes, and we wanted more involvement from the advocates’ perspective,” said Dale Eastman, vice president of the Alamo Breast Cancer Foundation.

“The centers represent a great opportunity to conduct transdisciplinary science to explore the problem of breast cancer from many different perspectives, including the valuable perspective of [breast cancer survivor] advocates,” said Robert Hiatt, principal investigator for the BCERC at the University of California, San Francisco, which collaborates with Lawrence Berkeley National Laboratory and serves as the coordinating center for the BCERC network. The other three centers are housed at the Fox Chase Cancer Center in Philadelphia (which collaborates with Mount Sinai School of Medicine in New York and the University of Alabama at Birmingham), the University of Cincinnati in Ohio (which collaborates with Cincinnati Children’s Hospital Medical Center), and Michigan State University in East Lansing.

Three important features characterize the BCERC program. First, the four centers work together as a network in which experimental methods are coordinated in order to maximize the pooling and comparison of the data generated. Second, to allow adequate time to track the subjects and collect comprehensive information on the onset and progress of puberty, the BCERCs will be funded for seven years, an unusually long time, given that most NIH grants are funded for a maximum of five years. Third, representatives of breast cancer survivor advocacy organizations are integral members of the centers. The advocate members participate in many aspects of the decision-making processes, collaborate with the Community Outreach and Translation Cores of the centers, and added much to the discussion at the annual meeting.

## Basic Biology Advances

Many of the significant advances of the past year were made in basic biology studies that use laboratory rodents and cell cultures as models. “This is not surprising, as the human studies are prospective and will yield their most valuable information over time,” said Les Reinlib, a program official at the NIEHS who directs the BCERC program.

At the November meeting, Deborah J. Clegg, an assistant professor of psychiatry at the University of Cincinnati, described the insights of her team into understanding the links between obesity, body fat distribution, and postmenopausal breast cancer. Based on her observations of how fat distributes in various body regions, Clegg hypothesized that women who accumulate fat in the upper body—the pattern typically seen in men—would have a greater breast cancer risk than women who accumulate fat in the lower body and thighs. Her team is the first to directly test this idea in humans. Clegg’s hypothesis is supported by reports linking insulin resistance, increased recurrence of tumors, and lower survival in obese breast cancer patients with the male pattern of fat distribution. Clegg suggested that body fat distribution may be a better measure than body mass index in determining breast cancer risk.

The team lead by Jose Russo, director of the Fox Chase Cancer Center BCERC and a senior member of the Fox Chase Medical Science Division, is studying the impact of endocrine disruptors on mammary gland development in rats. In this study, animals are exposed at different times in development to environmental pollutants of concern, including bisphenol A and butyl benzyl phthalate. Bisphenol A is an estrogenic substance that is used in the production of some plastics and in food container coatings, while butyl benzyl phthalate is used to plasticize polyvinyl chloride and other polymers. These investigators are finding that, depending on the time of exposure and the age of the young rats upon examination, different genes are up- or down-regulated by the exposures.

Their studies demonstrate that these compounds modify the genomic profile of the rat mammary gland and that these changes are age-specific, indicating windows of vulnerability in the development of the gland. “Interestingly, we are finding that some of these genes, such as glutamic decarboxylase 1, that are affected by exposure to estrogenic plasticizers have been implicated in other diseases such as autism, bipolar disorders, schizophrenia, diabetes, and cancer,” said Russo. “So there is an opportunity here to investigate not only how these compounds affect the genomic profile of the mammary gland but also how other organs are affected, explaining other diseases as well.”

At Michigan State University, center director and physiology professor Sandra Haslam is conducting studies in both rats and mice. Her work focuses on the study of the normal mammary gland in order to understand how progesterone, a hormone secreted in the second half of the menstrual cycle, is involved in breast development and perhaps in carcinogenesis. She said, “For prevention methods to be produced, we must understand the normal developmental process.” To do this, her team is defining the architecture and timing of two forms of progesterone receptors (PR-A and PR-B) as they are expressed on mammary gland cells from rats and mice. From the patterns of expression of these receptors and their colocalization with proliferation markers, Haslam infers the roles of the receptors on cell growth and maturation.

Haslam showed that the rat model is more similar than the mouse model to the human breast in terms of the patterns of receptors expressed. However, the mouse model exhibits novel differences that may allow a better understanding of the differences in the functional roles of PR-A and PR-B. Each animal model may provide information about certain aspects of the human condition but not others, given the many differences that exist among the species. Said Haslam, “Ideally, we would have access to human breast tissues from different times in development, but it is extremely difficult [to get breast tissue from women who do not have cancer].”

Human cells have been used in the laboratory to try to study some aspects of carcinogenesis that may be uniquely human and thus are difficult to study in rodents. Paul Yaswen, a staff scientist at the Lawrence Berkeley National Laboratory, is using human breast epithelial cells to define molecular events and cellular characteristics that allow tumor progression. When mammary cells become exposed to a carcinogen, their genomic tumor suppression pathways fail, and the cells become immortalized, or able to reproduce without proper controls. At the meeting, Yaswen presented work on two main tumor suppression pathways involved in blocking indefinite proliferation of breast cells derived from normal human tissue. These tumor suppression pathways represent a gauntlet that carcinogen-exposed cells must overcome in order to acquire cancerous properties.

## Listening and Learning

In the closing session, Gwen Collman, chief of the NIEHS Susceptibility and Population Health Branch, presented a framework upon which the results from the different BCERCs will fill in the gaps of scientific understanding about mammary gland development and breast cancer in the context of likely environmental carcinogens. “It was always our intention that [the BCERC program] would spark dialogue between laboratories, scientists of different disciplines, and advocates,” said Collman.

Although the data presented at the scientific sessions reflect advances in uncovering the link between environmental exposures and breast cancer, there’s still a long way to go before a cure for breast cancer is found. “We have been with the scientists from the beginning, and we have seen the progress, but what do we tell our communities of women who are dying of breast cancer right now?” asked Virginia Regnante, president of the West Islip Breast Cancer Coalition. She suggested that scientists should concentrate on testing the many chemicals that are likely to cause cancer in communities with high rates of breast cancer. “It is an inspiration to have the advocates not be afraid to tell us what they need from us scientists,” answered Irma Russo, an investigator in the Fox Chase BCERC.

## Figures and Tables

**Figure:**